# 2-Hy­droxy­ethyl­ammonium iodide

**DOI:** 10.1107/S1600536814009581

**Published:** 2014-05-03

**Authors:** Christina Kohrt, Anke Spannenberg, Thomas Werner

**Affiliations:** a Leibniz-Institut für Katalyse e. V. an der Universität Rostock, Albert-Einstein-Strasse 29a, 18059 Rostock, Germany

## Abstract

In the crystal structure of the title salt, C_2_H_8_NO^+^·I^−^, N—H⋯O, N—H⋯I and O—H⋯I hydrogen bonds lead to the formation of layers staggered along the *c* axis.

## Related literature   

A variety of compounds are known in the literature involving the cation [NH_3_CH_2_CH_2_OH]^+^. A WebCSD search (Release April 2014) yielded 85 examples (Thomas *et al.*, 2010 ▶), see for example: Koo *et al.* (1974 ▶) for 2-hy­droxy­ethyl­ammonium bromide, or Koo *et al.* (1972 ▶) for 2-hy­droxy­ethyl­ammonium chloride.
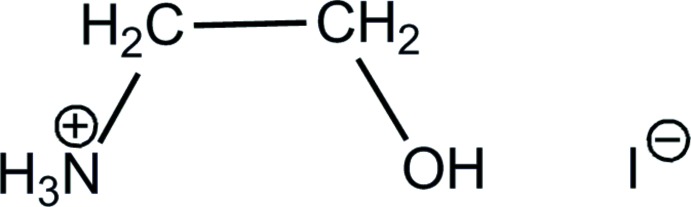


## Experimental   

### 

#### Crystal data  

C_2_H_8_NO^+^·I^−^
*M* *_r_* = 188.99
Triclinic, 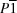

*a* = 4.6557 (4) Å
*b* = 7.5432 (6) Å
*c* = 8.1787 (7) Å
α = 85.235 (2)°
β = 78.091 (2)°
γ = 77.544 (2)°
*V* = 274.21 (4) Å^3^
*Z* = 2
Mo *K*α radiation
μ = 5.70 mm^−1^
*T* = 150 K
0.34 × 0.12 × 0.03 mm

#### Data collection  

Bruker Kappa APEXII DUO diffractometer
Absorption correction: multi-scan (*SADABS*; Bruker, 2008 ▶) *T*_min_ = 0.672, *T*_max_ = 0.843
4884 measured reflections
1319 independent reflections
1254 reflections with *I* > 2σ(*I*)
*R*_int_ = 0.021

#### Refinement  

*R*[*F* ^2^ > 2σ(*F* ^2^)] = 0.014
*wR*(*F* ^2^) = 0.032
*S* = 1.08
1319 reflections
48 parameters
H-atom parameters constrained
Δρ_max_ = 0.58 e Å^−3^
Δρ_min_ = −0.46 e Å^−3^

### 

Data collection: *APEX2* (Bruker, 2011 ▶); cell refinement: *SAINT* (Bruker, 2009 ▶); data reduction: *SAINT*; program(s) used to solve structure: *SHELXS97* (Sheldrick, 2008 ▶); program(s) used to refine structure: *SHELXL97* (Sheldrick, 2008 ▶); molecular graphics: *XP* in *SHELXTL* (Sheldrick, 2008 ▶); software used to prepare material for publication: *SHELXTL*.

## Supplementary Material

Crystal structure: contains datablock(s) I, Global. DOI: 10.1107/S1600536814009581/zl2586sup1.cif

Structure factors: contains datablock(s) I. DOI: 10.1107/S1600536814009581/zl2586Isup2.hkl

Supporting information file. DOI: 10.1107/S1600536814009581/zl2586Isup3.cml

CCDC reference: 999797

Additional supporting information:  crystallographic information; 3D view; checkCIF report

## Figures and Tables

**Table 1:** Hydrogen-bond geometry (Å, °)

*D*—H⋯*A*	*D*—H	H⋯*A*	*D*⋯*A*	*D*—H⋯*A*
N1—H1*A*⋯O1^i^	0.91	1.93	2.800 (2)	158
N1—H1*B*⋯I1^ii^	0.91	2.75	3.5825 (18)	152
N1—H1*C*⋯I1^iii^	0.91	2.78	3.6322 (18)	155
O1—H1*D*⋯I1	0.84	2.72	3.5100 (15)	157

Symmetry codes: (i) 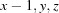; (ii) 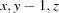; (iii) 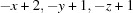.

**Fig. 1.**
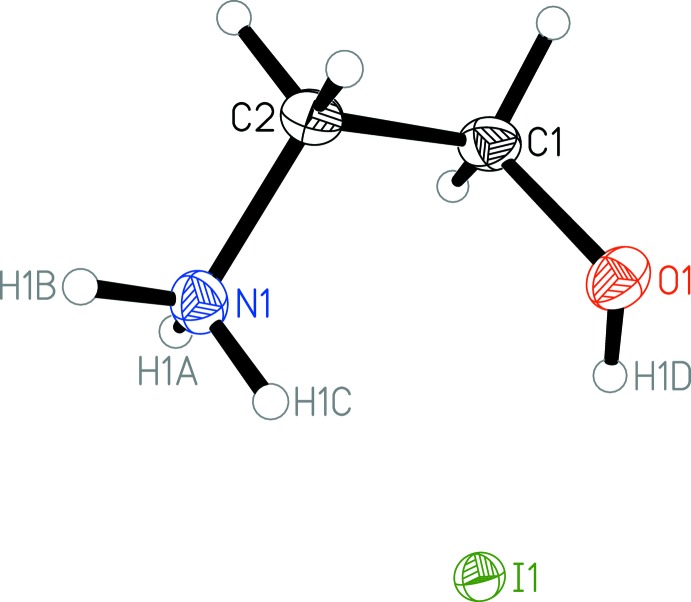
The molecular structure of the title compound in the crystal. Displacement ellipsoids are drawn at the 50% probability level.

**Fig. 2.**
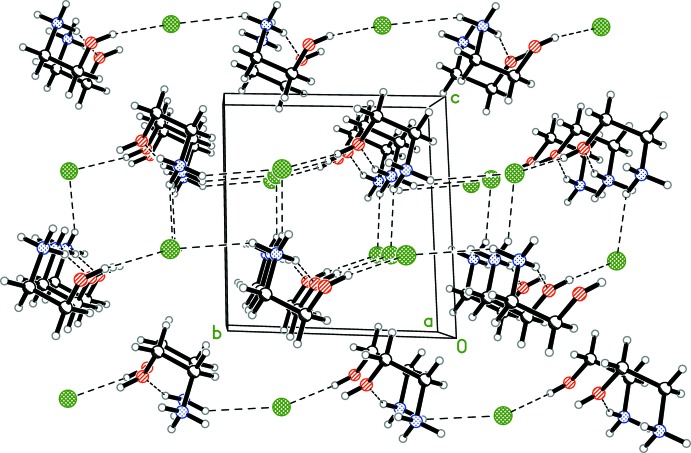
Packing plot; hydrogen bonds are shown as dashed lines.

## supplementary crystallographic information

### 1. Comment

Recently we were interested in the synthesis of perfluorinated organocatalysts. In this context we tried to alkylate 2-aminoethanol with 1*H*,1*H*,2*H*,2*H*-perfluorooctyliodide. Unfortunately we did not obtain the desired product under the chosen reaction conditions. However, instead we were able to isolate the title compound in excellent yield. The molecular structure of the ammonium iodide shows a nitrogen atom carrying three protons and one 2-hydroxyethyl-group and the iodide as anion (Fig. 1). The cations are aggregated through N—H···O hydrogen bonds in a linear arrangement parallel to the *a* axis. These chains are extended by N—H···I and O—H···I hydrogen bonds into layers staggered along the *c* axis (Fig. 2).

A variety of compounds involving the same cation had been reported in the literature. A WebCSD search (Release April 2014, Thomas *et al.* (2010)) yielded 85 examples of hydroxyethylammonium salts; for the bromide and chloride salts most closely related to the iodide title compound, please see Koo *et al.* (1974) and Koo *et al.* (1972), respectively.

### 2. Experimental

2-Aminoethanol (4.09 mmol, 250 mg, 1 eq) was added to 1*H*,1*H*,2*H*,2*H*-perfluorooctyliodide (12.3 mmol, 5.80 g, 3 eq) in a pressure pipe under argon. The solution was stirred at 80°C for 24 h. Afterwards the resulting yellow solution was layered with 2,2,2-trifluoroethanol and crystals precipitated directly from the mixture. 84% (3.43 mmol, 649 mg) of the title compound were obtained as colorless crystals. ^1^H NMR (CF_3_—CD_2_—OD): *δ* 3.59–3.48 (br m, 2H); 2.78–2.67 (br m, 2H) ppm. ^13^C NMR (CF_3_—CD_2_—OD): *δ* 62.47 (s, CH_2_); 44.03 (s, CH_2_) ppm. Elemental analysis calculated (%) for C_2_H_8_INO: C 12.71, H 4.27, N 7.41; found: C 12.97, H 4.10, N 7.48.

### 3. Refinement

H1A - H1D were clearly identified in difference Fourier maps. All H atoms were placed in idealized positions with d(O—H) = 0.84, d(N—H) = 0.91, d(C—H) = 0.99 Å and refined using a riding model with *U*_iso_(H) fixed at 1.2 *U*_eq_(C) and 1.5 *U*_eq_(N, O)

### Crystal data

**Table:**

C_2_H_8_NO^+^·I^−^	*Z* = 2
*M**_r_* = 188.99	*F*(000) = 176
Triclinic, *P*1	*D*_x_ = 2.289 Mg m^−^^3^
*a* = 4.6557 (4) Å	Mo *K*α radiation, λ = 0.71073 Å
*b* = 7.5432 (6) Å	Cell parameters from 3767 reflections
*c* = 8.1787 (7) Å	θ = 2.8–29.0°
α = 85.235 (2)°	µ = 5.70 mm^−^^1^
β = 78.091 (2)°	*T* = 150 K
γ = 77.544 (2)°	Plate, colorless
*V* = 274.21 (4) Å^3^	0.34 × 0.12 × 0.03 mm

### Data collection

**Table:**

Bruker Kappa APEXII DUO diffractometer	1319 independent reflections
Radiation source: fine-focus sealed tube	1254 reflections with *I* > 2σ(*I*)
Curved graphite monochromator	*R*_int_ = 0.021
Detector resolution: 8.3333 pixels mm^-1^	θ_max_ = 28.0°, θ_min_ = 2.6°
φ and ω scans	*h* = −6→6
Absorption correction: multi-scan (*SADABS*; Bruker, 2008)	*k* = −9→9
*T*_min_ = 0.672, *T*_max_ = 0.843	*l* = −10→10
4884 measured reflections	

### Refinement

**Table:**

Refinement on *F*^2^	Primary atom site location: structure-invariant direct methods
Least-squares matrix: full	Secondary atom site location: difference Fourier map
*R*[*F*^2^ > 2σ(*F*^2^)] = 0.014	Hydrogen site location: inferred from neighbouring sites
*wR*(*F*^2^) = 0.032	H-atom parameters constrained
*S* = 1.08	*w* = 1/[σ^2^(*F*_o_^2^) + (0.0163*P*)^2^ + 0.0136*P*] where *P* = (*F*_o_^2^ + 2*F*_c_^2^)/3
1319 reflections	(Δ/σ)_max_ = 0.002
48 parameters	Δρ_max_ = 0.58 e Å^−^^3^
0 restraints	Δρ_min_ = −0.46 e Å^−^^3^

### Special details

**Table:**

Geometry. All e.s.d.'s (except the e.s.d. in the dihedral angle between two l.s. planes) are estimated using the full covariance matrix. The cell e.s.d.'s are taken into account individually in the estimation of e.s.d.'s in distances, angles and torsion angles; correlations between e.s.d.'s in cell parameters are only used when they are defined by crystal symmetry. An approximate (isotropic) treatment of cell e.s.d.'s is used for estimating e.s.d.'s involving l.s. planes.
Refinement. Refinement of *F*^2^ against ALL reflections. The weighted *R*-factor *wR* and goodness of fit *S* are based on *F*^2^, conventional *R*-factors *R* are based on *F*, with *F* set to zero for negative *F*^2^. The threshold expression of *F*^2^ > σ(*F*^2^) is used only for calculating *R*-factors(gt) *etc*. and is not relevant to the choice of reflections for refinement. *R*-factors based on *F*^2^ are statistically about twice as large as those based on *F*, and *R*- factors based on ALL data will be even larger.

### Fractional atomic coordinates and isotropic or equivalent isotropic displacement parameters (Å^2^)

**Table:**

	*x*	*y*	*z*	*U*_iso_*/*U*_eq_	
I1	0.63581 (3)	0.752733 (16)	0.669449 (16)	0.01732 (5)	
O1	1.1624 (3)	0.3874 (2)	0.79532 (19)	0.0204 (3)	
H1D	1.0844	0.4856	0.7510	0.031*	
N1	0.7519 (4)	0.2059 (2)	0.6780 (2)	0.0182 (4)	
H1A	0.5779	0.2898	0.7009	0.027*	
H1B	0.7164	0.1061	0.6366	0.027*	
H1C	0.8882	0.2542	0.6011	0.027*	
C1	0.9351 (5)	0.3140 (3)	0.9069 (3)	0.0189 (4)	
H1E	0.7493	0.4085	0.9290	0.023*	
H1F	1.0004	0.2765	1.0146	0.023*	
C2	0.8719 (5)	0.1527 (3)	0.8344 (3)	0.0182 (4)	
H2A	1.0590	0.0596	0.8098	0.022*	
H2B	0.7245	0.0988	0.9174	0.022*	

### Atomic displacement parameters (Å^2^)

**Table:**

	*U*^11^	*U*^22^	*U*^33^	*U*^12^	*U*^13^	*U*^23^
I1	0.01634 (8)	0.01767 (8)	0.01768 (8)	−0.00360 (5)	−0.00336 (5)	0.00129 (5)
O1	0.0182 (7)	0.0167 (7)	0.0249 (8)	−0.0035 (6)	−0.0021 (6)	0.0012 (6)
N1	0.0164 (9)	0.0213 (9)	0.0175 (9)	−0.0048 (7)	−0.0027 (7)	−0.0022 (7)
C1	0.0218 (11)	0.0203 (11)	0.0139 (10)	−0.0046 (8)	−0.0020 (8)	0.0000 (8)
C2	0.0188 (10)	0.0176 (11)	0.0182 (10)	−0.0023 (8)	−0.0058 (8)	0.0018 (8)

### Geometric parameters (Å, º)

**Table:**

O1—C1	1.425 (3)	C1—C2	1.505 (3)
O1—H1D	0.8400	C1—H1E	0.9900
N1—C2	1.490 (3)	C1—H1F	0.9900
N1—H1A	0.9100	C2—H2A	0.9900
N1—H1B	0.9100	C2—H2B	0.9900
N1—H1C	0.9100		
			
C1—O1—H1D	109.5	O1—C1—H1F	109.5
C2—N1—H1A	109.5	C2—C1—H1F	109.5
C2—N1—H1B	109.5	H1E—C1—H1F	108.0
H1A—N1—H1B	109.5	N1—C2—C1	111.23 (16)
C2—N1—H1C	109.5	N1—C2—H2A	109.4
H1A—N1—H1C	109.5	C1—C2—H2A	109.4
H1B—N1—H1C	109.5	N1—C2—H2B	109.4
O1—C1—C2	110.92 (17)	C1—C2—H2B	109.4
O1—C1—H1E	109.5	H2A—C2—H2B	108.0
C2—C1—H1E	109.5		
			
O1—C1—C2—N1	−63.2 (2)		

### Hydrogen-bond geometry (Å, º)

**Table:**

*D*—H···*A*	*D*—H	H···*A*	*D*···*A*	*D*—H···*A*
N1—H1*A*···O1^i^	0.91	1.93	2.800 (2)	158
N1—H1*B*···I1^ii^	0.91	2.75	3.5825 (18)	152
N1—H1*C*···I1^iii^	0.91	2.78	3.6322 (18)	155
O1—H1*D*···I1	0.84	2.72	3.5100 (15)	157

Symmetry codes: (i) *x*−1, *y*, *z*; (ii) *x*, *y*−1, *z*; (iii) −*x*+2, −*y*+1, −*z*+1.
